# Association of systemic inflammatory indices with gastrointestinal tumors: evidence from NHANES and a hospital-based cohort

**DOI:** 10.3389/fmed.2026.1886964

**Published:** 2026-08-10

**Authors:** Rui Luo, Haifen Ji, Jiwen Pan, Mingzhu Wei, Dongmei Gao

**Affiliations:** 1 Department of Clinical Laboratory, The First People's Hospital of Hefei, Anhui, China; 2 Medical Pathology Center, Hefei Cancer Hospital, Chinese Academy of Sciences, Anhui, China

**Keywords:** gastrointestinal tumors, hospital-based cohort, inflammatory indices, NHANES, systemic inflammation

## Abstract

**Background:**

Systemic inflammation plays an important role in tumor development and progression. Several inflammation-related indices derived from routine blood tests have been proposed as potential biomarkers for cancer. However, their associations with gastrointestinal tumors in large population-based datasets remain insufficiently explored.

**Methods:**

Data from the National Health and Nutrition Examination Survey (NHANES) were used to examine the associations between nine systemic inflammatory indices and gastrointestinal tumors. Multivariable logistic regression, propensity score matching, and restricted cubic spline analyses were performed. Mortality-linked NHANES data were further analyzed using Cox regression and Kaplan–Meier methods. An independent hospital-based cohort was used to validate the main findings.

**Results:**

Several systemic inflammatory indices were significantly associated with gastrointestinal tumor status in NHANES. NLR and RAR showed particularly strong associations and ranked among the most influential variables in the machine-learning analyses. Restricted cubic spline analyses indicated nonlinear association patterns, with higher NLR and RAR levels corresponding to greater odds of gastrointestinal tumors. The main findings remained generally consistent in the propensity score-matched cohort. In the hospital-based cohort, patients with gastrointestinal tumors also showed higher systemic inflammatory levels than healthy controls. A nomogram incorporating CALLY, NLR, RAR, and clinical variables demonstrated good discriminative performance, with an AUC of 0.852. In addition, several baseline inflammatory indices were independently associated with all-cause mortality among patients with gastrointestinal tumors.

**Conclusion:**

Systemic inflammatory indices derived from routine blood tests were associated with gastrointestinal tumor status and all-cause mortality. These main findings were further supported by the hospital-based cohort. Routine inflammation-related indices may therefore provide useful information for identifying tumor-associated inflammatory changes and evaluating prognosis in patients with gastrointestinal tumors.

## 1 Introduction

Gastrointestinal tumors (GIT) are among the most common malignancies worldwide and represent a major public health challenge (1–3). These cancers originate from the gastrointestinal tract, including the esophagus, stomach, and colorectum, which are among the most commonly affected sites. Together, they account for a large proportion of global cancer incidence and mortality (4). Although substantial progress has been made in screening and treatment, many patients are still diagnosed at advanced stages, which limits the effectiveness of therapeutic interventions (5). Therefore, identifying accessible biomarkers that can help recognize individuals at increased risk remains an important priority (6, 7).

Systemic inflammation is increasingly recognized as a critical factor in tumor development and progression (8, 9). Chronic inflammatory responses can promote carcinogenesis through multiple mechanisms, including immune dysregulation, oxidative stress, angiogenesis, and remodeling of the tumor microenvironment (10, 11). In recent years, several inflammation-related indices derived from routine blood tests have been proposed as surrogate markers reflecting systemic inflammatory status. These indices include the neutrophil-to-lymphocyte ratio (NLR), platelet-to-lymphocyte ratio (PLR), lymphocyte-to-monocyte ratio (LMR), and other composite indicators (12–14). Because these markers are inexpensive and widely available in clinical practice, they have attracted growing attention as potential biomarkers for cancer risk assessment.

However, most previous studies have primarily focused on single inflammatory indicators, which may not fully capture the complex and dynamic nature of systemic inflammation. In reality, inflammatory responses involve multiple interacting pathways (15), and different indices may reflect distinct aspects of immune and inflammatory status (16). Therefore, relying on a single marker may lead to incomplete or inconsistent conclusions. A comprehensive evaluation integrating multiple inflammatory indices in relation to digestive system cancers remains limited. In addition, the potential dose–response relationships between inflammatory indices and cancer status have not been sufficiently characterized, particularly in large population-based settings. Clarifying the strength and shape of these associations, including potential nonlinear patterns, may improve the identification of individuals with inflammation-related features associated with GIT.

In this study, we used data from the National Health and Nutrition Examination Survey (NHANES) to investigate the associations between nine systemic inflammatory indices and GIT status. In addition, a clinical model integrating inflammatory indices and baseline characteristics was constructed to distinguish individuals with cancer from healthy controls.

## 2 Methods

### 2.1 Study design, data source, and study population

This study was conducted using a two-cohort design, including a population-based dataset and an independent hospital-based cohort. The population-based analysis was performed using data from NHANES, a nationally representative survey of the civilian, noninstitutionalized population in the United States. NHANES collects extensive demographic, clinical, and laboratory data through standardized interviews, physical examinations, and laboratory tests. Participants with available data on inflammatory markers, cancer status, and relevant covariates were included in the present study, while individuals with missing key variables were excluded.

In addition, an independent hospital-based cohort was established at The First People's Hospital of Hefei to externally validate the findings from the NHANES analysis. Consecutive adult patients (≥18 years) diagnosed with digestive system cancers between January 2023 and December 2025 were retrospectively enrolled. The cohort included patients with gastrointestinal tract tumors (esophageal, gastric, colon, and rectal cancers) as well as hepatobiliary–pancreatic tumors (liver, gallbladder, and pancreatic cancers). Eligible participants were required to have complete demographic information and laboratory measurements for the calculation of inflammatory indices. Patients with concurrent acute infections, autoimmune diseases, hematological disorders, other active malignancies, or incomplete clinical or laboratory data were excluded to minimize potential influences on systemic inflammatory markers.

Healthy controls were recruited from individuals aged ≥18 years who underwent routine health examinations during the same period and had no history of malignant tumors. Because this was a retrospective observational study, all eligible participants meeting the predefined inclusion criteria during the study period were consecutively enrolled, and no formal sample size calculation was performed. Owing to the retrospective design, detailed information regarding recurrence status, metastatic disease, and previous antitumor treatment was not consistently available for all patients.

All NHANES data are publicly available and were collected with approval from the National Center for Health Statistics Research Ethics Review Board, and written informed consent was obtained from all participants. The hospital-based study was approved by the Ethics Committee of The First People's Hospital of Hefei (Approval No. 2026-156-01) and was conducted in accordance with the Declaration of Helsinki and relevant institutional ethical standards.

### 2.2 Definition of gastrointestinal tumors

In the NHANES dataset, GIT was identified based on the self-reported medical conditions questionnaire. Participants reporting cancers of the esophagus, stomach, colon, or rectum were classified as having GIT. In the hospital-based cohort, tumor diagnoses were confirmed from clinical and pathological records.

### 2.3 Assessment of inflammatory indices

Systemic inflammatory indices were calculated using peripheral blood parameters according to the following formulas:


$$\begin{array}{l} NLR\;=\;Neutrophil\;count\frac{\left(\frac{10^{9}}{L}\right)}{Lymphocyte\;count\;\left(\frac{10^{9}}{L}\right)}\\ PLR\;=\;Platelet\;count\frac{\left(\frac{10^{9}}{L}\right)}{Lymphocyte\;count\;\left(\frac{10^{9}}{L}\right)}\\ LMR\;=\;Lymphocyte\;count\frac{\left(\frac{10^{9}}{L}\right)}{Monocyte\;count\;\left(\frac{10^{9}}{L}\right)}\\ SIRI\;=\;Neutrophil\;count\;\left(\frac{10^{9}}{L}\right)\\ \times \;Monocyte\;count\frac{\left(\frac{10^{9}}{L}\right)}{Lymphocyte\;count\;\left(\frac{10^{9}}{L}\right)}\\ SII\;=\;Platelet\;count\;\left(\frac{10^{9}}{L}\right)\\ \times \;Neutrophil\;count\frac{\left(\frac{10^{9}}{L}\right)}{Lymphocyte\;count\;\left(\frac{10^{9}}{L}\right)}\\ PIV\;=\;Platelet\;count\;\left(\frac{10^{9}}{L}\right)\times \;Neutrophil\;count\;\left(\frac{10^{9}}{L}\right)\\ \times \;Monocyte\;count\frac{\left(\frac{10^{9}}{L}\right)}{Lymphocyte\;count\;\left(\frac{10^{9}}{L}\right)}\\ RAR\;=\;\frac{RDW\left(\%\right)}{Albumin\;\left(\frac{g}{L}\right)}\\ NAR\;=\;Neutrophil\;count\frac{\left(\frac{10^{9}}{L}\right)}{Albumin\;\left(\frac{g}{L}\right)}\\ CALLY\;index\;=\;Albumin\;\left(\frac{g}{L}\right)\times \;\frac{Lymphocyte\;count\left(\frac{10^{9}}{L}\right)}{CRP\;\left(\frac{mg}{L}\right)} \end{array}$$

### 2.4 Covariates

Potential confounders were selected based on previous studies and data availability in NHANES. These included demographic variables (age, sex, and race/ethnicity), socioeconomic indicators (education level and poverty income ratio, PIR), lifestyle factors (smoking status and alcohol consumption), body mass index (BMI), and comorbidities (diabetes and hypertension). These variables were included as covariates in the multivariable analyses of the NHANES cohort. For the hospital-based validation cohort, age and sex were included as adjustment variables because detailed information on race/ethnicity, socioeconomic status, lifestyle factors, BMI, and comorbidities was not consistently available. Therefore, the hospital-based cohort was used primarily to validate the direction and consistency of the associations observed in NHANES rather than to fully replicate the covariate-adjusted NHANES models.

### 2.5 Machine-learning analysis

To evaluate the predictive value of inflammatory indices for gastrointestinal tumors, nine different machine-learning algorithms were developed using the inflammatory markers as input variables. The dataset was randomly divided into a training set and a validation set. During model construction, standardized model selection and overfitting prevention strategies were applied. All models were trained and evaluated using five-fold stratified cross-validation, and model performance was assessed using receiver operating characteristic (ROC) curves and the area under the curve (AUC). In addition, SHapley Additive exPlanations (SHAP) analysis was performed to quantify the contribution of each inflammatory index to model prediction and to rank feature importance. To further assess the potential influence of class imbalance, a sensitivity analysis was performed using a balanced 1:1 dataset consisting of all 191 participants with GIT and 191 randomly selected controls. The same Boosting model was then repeated in this balanced dataset to further validate the relative importance of inflammation-related variables.

### 2.6 Multivariable logistic regression analysis

Logistic regression was used to assess the associations between inflammatory indices and gastrointestinal tumors. Each index was divided into quartiles, with the lowest quartile as the reference. Three models were fitted: Model 1 was unadjusted; Model 2 adjusted for age, sex, and race/ethnicity; and Model 3 was further adjusted for education level, poverty income ratio (PIR), smoking status, alcohol consumption, BMI, diabetes, and hypertension. Results were reported as odds ratios (ORs) with 95% confidence intervals (CIs). In addition, propensity score matching (PSM) was performed to reduce the imbalance between controls and participants with GIT. PSM was conducted using the “MatchIt” package in *R* at a 1:4 ratio. Covariate balance was assessed using standardized mean differences (SMDs), with SMD < 0.05 indicating good balance. Logistic regression analyses were then repeated in the matched cohort using the “survey” package in *R* to evaluate the associations between the nine inflammatory indices and gastrointestinal tumor status.

### 2.7 Subgroup and dose–response analyses

Stratified analyses were conducted according to sex, age, smoking status, and alcohol consumption to explore whether the associations between inflammatory indices and gastrointestinal tumor status differed across population subgroups. Because the number of GIT cases in NHANES was limited, especially after stratification, no additional covariate adjustment was performed in the subgroup analyses to avoid model overfitting and unstable estimates. Restricted cubic spline (RCS) analyses were further used to explore potential dose–response relationships between inflammatory indices and gastrointestinal tumor status. The RCS models were adjusted for the same covariates as Model 3, including age, sex, race/ethnicity, education level, poverty income ratio, smoking status, alcohol consumption, BMI, diabetes, and hypertension. Both overall and nonlinear associations were assessed.

### 2.8 Construction of the composite inflammation score

Five representative markers (RAR, NLR, PLR, LMR, and NAR) were selected for further analysis. Principal component analysis (PCA) was then applied to these indices, and the first principal component (PC1), which captured the largest proportion of variance, was extracted as the composite inflammation score.

### 2.9 Nomogram construction and evaluation

A nomogram was developed using selected inflammatory indices to distinguish individuals with GIT from controls. Model performance was evaluated using ROC analysis and calibration curves. The Hosmer–Lemeshow test was used to assess the agreement between predicted and observed outcomes.

### 2.10 Construction of a multivariable Cox proportional hazards regression model

Survival information was extracted for the 191 participants with GIT in NHANES. Among these patients, 121 deaths and 70 censored participants were identified during follow-up. The outcome was all-cause mortality. Each inflammatory index was categorized into quartiles. The proportional hazards assumption was first assessed for each inflammatory index. Variables satisfying the proportional hazards assumption were then evaluated using univariable Cox proportional hazards regression models.

Inflammatory indices with *P* < 0.05 in the univariable Cox regression analyses were further included in multivariable Cox proportional hazards regression models with progressive adjustment for covariates. Model 2 was adjusted for age, sex, race/ethnicity, education level, and poverty income ratio. Model 3 was further adjusted for smoking status, alcohol consumption, BMI, diabetes, and hypertension. Hazard ratios (HRs) and 95% CIs were calculated. A two-sided *P* < 0.05 was considered statistically significant.

### 2.11 Kaplan-Meier survival analysis

Survival information was extracted for the 191 participants with GIT in NHANES. Kaplan-Meier survival curve analysis was performed to compare survival differences across quartile groups of the nine inflammatory indices. The log-rank test was conducted using the “survival” package in *R* to evaluate differences in cumulative survival probabilities among different quartile groups. A two-sided *P* < 0.05 was considered statistically significant.

### 2.12 Statistical analysis

All statistical analyses were performed using R software (version 4.4.1; R Foundation for Statistical Computing, Vienna, Austria). Normally distributed continuous variables were expressed as mean ± standard deviation (SD) and compared using Student's *t*-test, whereas non-normally distributed variables were presented as median (interquartile range) and compared using the Mann–Whitney *U*-test. Categorical variables were summarized as counts and percentages and compared using the chi-square test. All tests were two-sided, and *P* < 0.05 was considered statistically significant.

## 3 Results

### 3.1 Baseline characteristics and distribution of inflammatory indices

A total of 21,521 participants were included in the NHANES analysis, comprising 21,330 controls and 191 individuals with GIT. The baseline demographic and clinical characteristics of the study population are presented in Table 1. Compared with controls, participants with GIT were significantly older, with the majority of cases occurring in individuals aged >50 years (91.6% vs. 42.5%). The distribution of race also differed between groups, with a higher proportion of non-Hispanic White participants among GIT cases. In addition, marital status and smoking status showed significant differences between groups, whereas no significant differences were observed for education level, alcohol consumption, BMI, or PIR. Regarding comorbidities, the prevalence of diabetes and hypertension was markedly higher among individuals with GIT than among controls.

**Table 1:** Baseline demographic and clinical characteristics and inflammatory indices according to GIT status.

Variable	Level	Control	GIT	*P*
*n*		21,330	191	
Gender (%)	Female	10,871 (51.0)	88 (46.1)	0.203
	Male	10,459 (49.0)	103 (53.9)	
Age (%)	>50	9,062 (42.5)	175 (91.6)	< 0.001
	≤ 50	12,268 (57.5)	16 (8.4)	
Race (%)	Mexican American	4,510 (21.1)	16 (8.4)	< 0.001
	Non-hispanic black	4,087 (19.2)	30 (15.7)	
	Non-hispanic white	10,502 (49.2)	135 (70.7)	
	Other hispanic	1,390 (6.5)	7 (3.7)	
	Other race	841 (3.9)	3 (1.6)	
Education (%)	9–11th gradess	3,492 (16.4)	35 (18.3)	0.086
	College graduate or above	4,235 (19.9)	27 (14.1)	
	High school grad	5,030 (23.6)	44 (23.0)	
	< 9th grade	2,734 (12.8)	35 (18.3)	
	Some college	5,839 (27.4)	50 (26.2)	
PIR (mean [SD])		2.60 (1.62)	2.45 (1.51)	0.221
Marry (%)	Divorced	2,115 (9.9)	27 (14.1)	< 0.001
	Living with partner	1,558 (7.3)	3 (1.6)	
	Married	11,792 (55.3)	98 (51.3)	
	Never married	3,506 (16.4)	7 (3.7)	
	Separated	679 (3.2)	6 (3.1)	
	Widowed	1,680 (7.9)	50 (26.2)	
Smoke (%)	No	11,201 (52.5)	72 (37.7)	< 0.001
	Yes	10,129 (47.5)	119 (62.3)	
Alcohol (%)	No	6,294 (29.5)	60 (31.4)	0.62
	Yes	15,036 (70.5)	131 (68.6)	
BMI (mean [SD])		28.79 (6.55)	28.58 (5.67)	0.669
Diabetes (%)	Borderline	331 (1.6)	4 (2.1)	< 0.001
	No	18,892 (88.6)	144 (75.4)	
	Yes	2,107 (9.9)	43 (22.5)	
Hypertension (%)	No	14,685 (68.8)	64 (33.5)	< 0.001
	Yes	6,645 (31.2)	127 (66.5)	
SIRI		1.04 (0.71, 1.52)	1.34 (0.83, 2.00)	< 0.001
CALLY		4.07 (1.69, 10.39)	3.00 (1.13, 7.57)	< 0.001
RAR		0.30 (0.28, 0.32)	0.31 (0.29, 0.35)	< 0.001
NLR		2.00 (1.50, 2.65)	2.33 (1.71, 3.20)	< 0.001
PLR		125.83 (100.00, 158.42)	136.25 (107.98, 177.25)	< 0.001
LMR		4.00 (3.00, 5.00)	3.12 (2.33, 4.00)	< 0.001
SII		508.27 (362.46, 714.77)	560.64 (375.30, 759.26)	0.036
PIV		265.42 (173.87, 408.77)	336.39 (171.94, 494.98)	0.004
NAR		0.10 (0.07, 0.12)	0.10 (0.07, 0.12)	0.298

Several inflammation-related biomarkers exhibited significant differences between the two groups. Participants with GIT had higher median levels of SIRI, RAR, NLR, PLR, SII, and PIV than controls, indicating a more pronounced systemic inflammatory state in the GIT group. In contrast, In contrast, the median LMR level was lower in participants with GIT than in controls, suggesting a relative reduction of lymphocyte-related immune responses in tumor patients. Overall, these findings indicate that multiple systemic inflammatory markers are markedly dysregulated in individuals with gastrointestinal tumors compared with healthy controls.

### 3.2 Machine-learning model performance and feature importance

**Figure 1:**
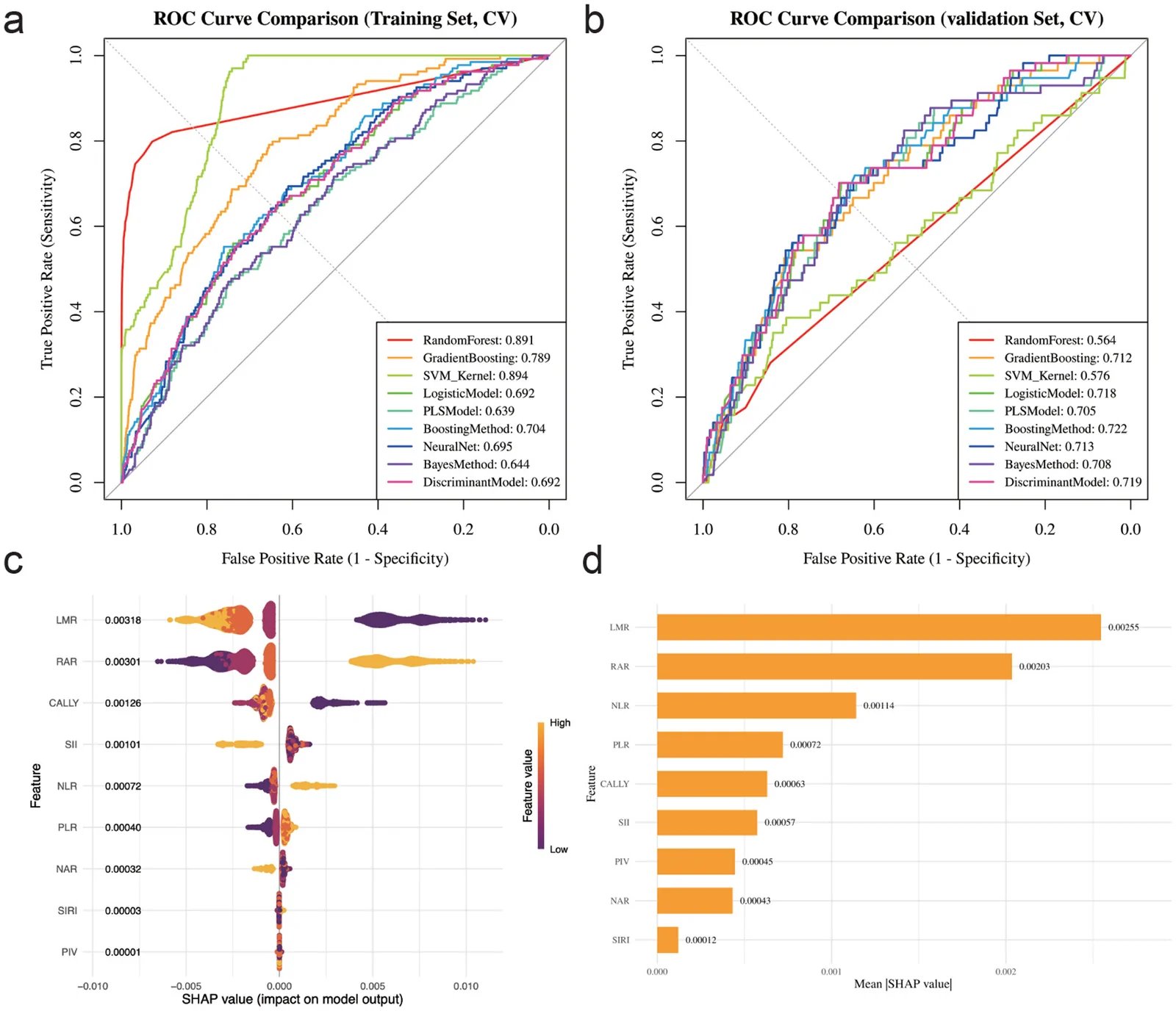
Machine-learning model performance and feature importance of inflammatory indices for gastrointestinal tumor prediction. **(a)** ROC curves of multiple machine-learning models in the training set. **(b)** ROC curves of the models in the validation set. **(c)** SHAP summary plot showing the impact of each inflammatory index on model prediction. **(d)** Ranking of feature importance based on mean SHAP values.

Multiple machine-learning algorithms were applied to evaluate the predictive ability of inflammatory indices for gastrointestinal tumors. In the training set, several models demonstrated good discriminative performance, with the Random Forest and Support Vector Machine models achieving the highest AUC values (Figure 1a). In the validation set, although the overall predictive performance slightly decreased, several models still maintained acceptable discrimination, with the boosting model showing the highest AUC (Figure 1b). To further explore the contribution of each inflammatory index to model prediction, SHAP analysis was performed. The results indicated that LMR, RAR, and NLR were the most influential variables, suggesting that these markers play a key role in distinguishing gastrointestinal tumor patients from healthy controls (Figures 1c–d). In addition, the sensitivity analysis based on a balanced 1:1 matched sample showed that inflammatory indices also retained important predictive value for gastrointestinal tumors, as reflected by the machine-learning model performance and SHAP-based feature importance analysis (Supplementary Figure 1).

### 3.3 Association between inflammatory indices and GIT

**Figure 2:**
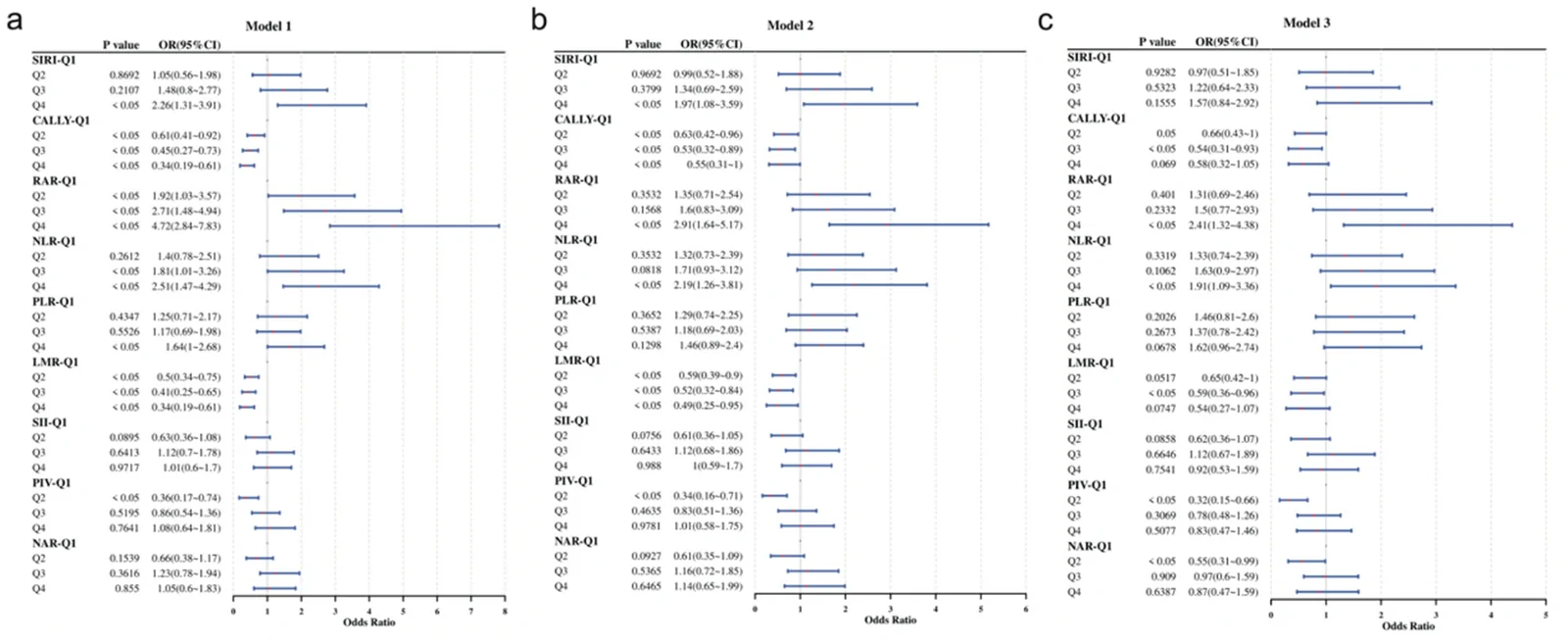
Association between inflammatory indices and gastrointestinal tumors based on multivariable logistic regression models. **(a)** Model 1 results without adjustment. **(b)** Model 2 results adjusted for demographic variables. **(c)** Model 3 results further adjusted for lifestyle factors and comorbidities.

Multivariable logistic regression analyses were conducted to evaluate the associations between inflammatory indices and gastrointestinal tumors across three progressively adjusted models (Figure 2). In Model 1, higher quartiles of several inflammatory markers, including SIRI, RAR, NLR, and PLR, were associated with increased odds of gastrointestinal tumors, whereas CALLY and LMR showed an inverse association with gastrointestinal tumor status. After additional adjustment for demographic and lifestyle factors in Model 2, these associations remained generally consistent, with RAR, NLR, and SIRI continuing to show positive associations with gastrointestinal tumor status, while CALLY and LMR maintained inverse associations. In the fully adjusted Model 3, similar trends persisted. Participants in the highest quartiles of RAR and NLR had significantly greater odds of GIT, whereas higher CALLY levels remained inversely associated with GIT status. Overall, these results suggest that multiple systemic inflammatory indices are independently associated with the occurrence of gastrointestinal tumors.

Notably, the results based on the balanced cohort generated by PSM were largely consistent with those obtained from the overall sample. CALLY, RAR, NLR and LMR remained associated with gastrointestinal tumor status, and these associations persisted across multiple covariate-adjusted models (Supplementary Figure 2). Exploratory subgroup analyses stratified by sex, age, smoking status, and alcohol consumption showed broadly similar crude association patterns for CALLY, LMR, NLR, and RAR across different population subgroups (Supplementary Figure 3).

### 3.4 Dose–response relationships between inflammatory indices and GIT

**Figure 3:**
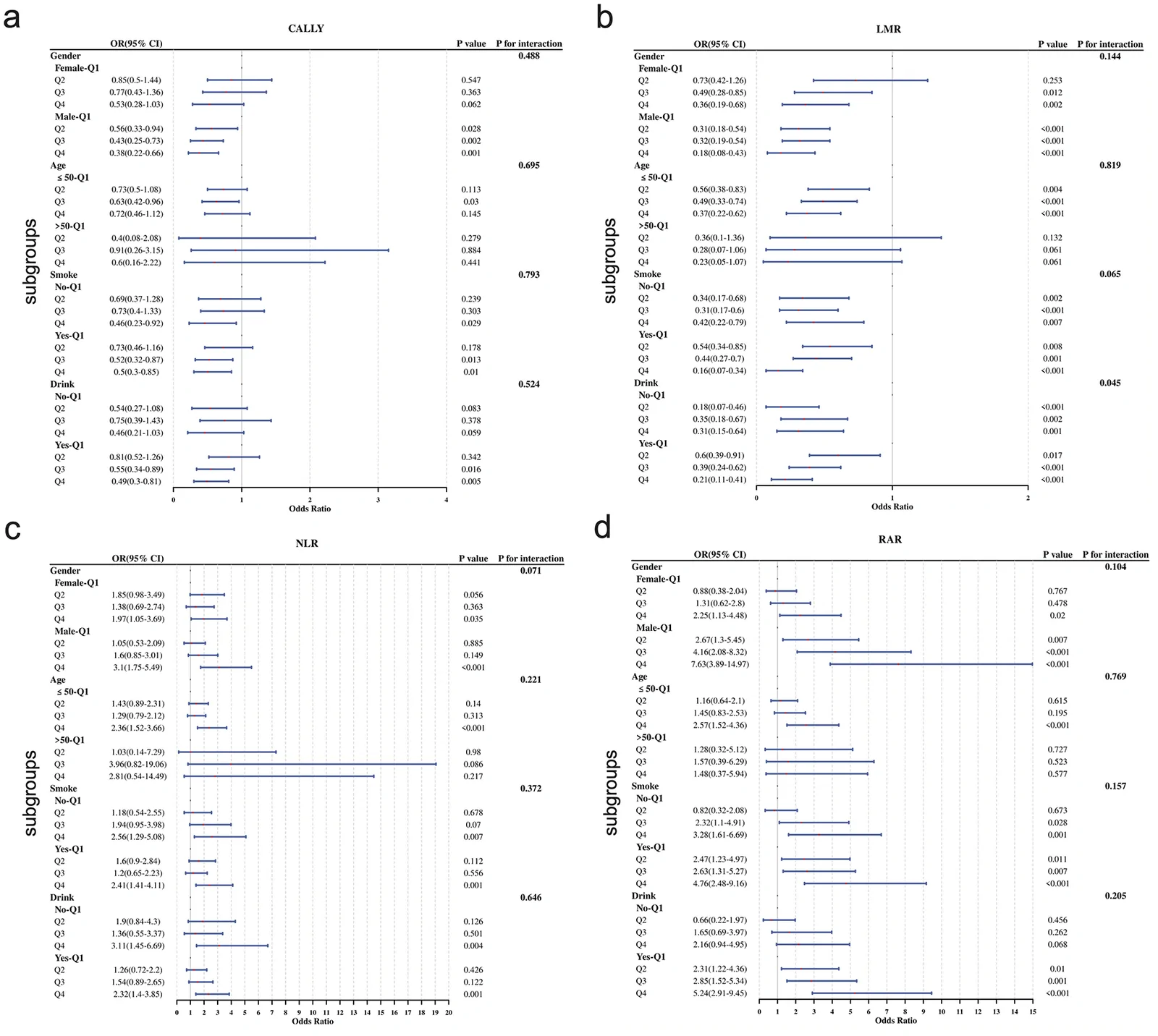
Dose–response relationships between inflammatory indices and gastrointestinal tumor status. Restricted cubic spline analyses showing the dose–response relationships between inflammatory indices and gastrointestinal tumors. Models were adjusted for the same covariates as Model 3, including age, sex, race/ethnicity, education level, poverty income ratio, smoking status, alcohol consumption, BMI, diabetes, and hypertension. **(a)** CALLY, **(b)** LMR, **(c)** NLR, and **(d)** RAR.

RCS analyses were performed to examine the dose–response patterns between four key inflammatory indices and GIT status (Figure 3). CALLY showed a significant inverse association, with the odds of GIT decreasing as CALLY levels increased and no evidence of nonlinearity (Figure 3a). LMR exhibited a nonlinear association with GIT status (Figure 3b). NLR and RAR showed significant positive nonlinear associations, with higher levels corresponding to greater odds of GIT (Figures 3c, d). Overall, these findings indicate distinct linear and nonlinear association patterns between systemic inflammatory status and GIT.

### 3.5 Construction and performance of the nomogram model

**Figure 4:**
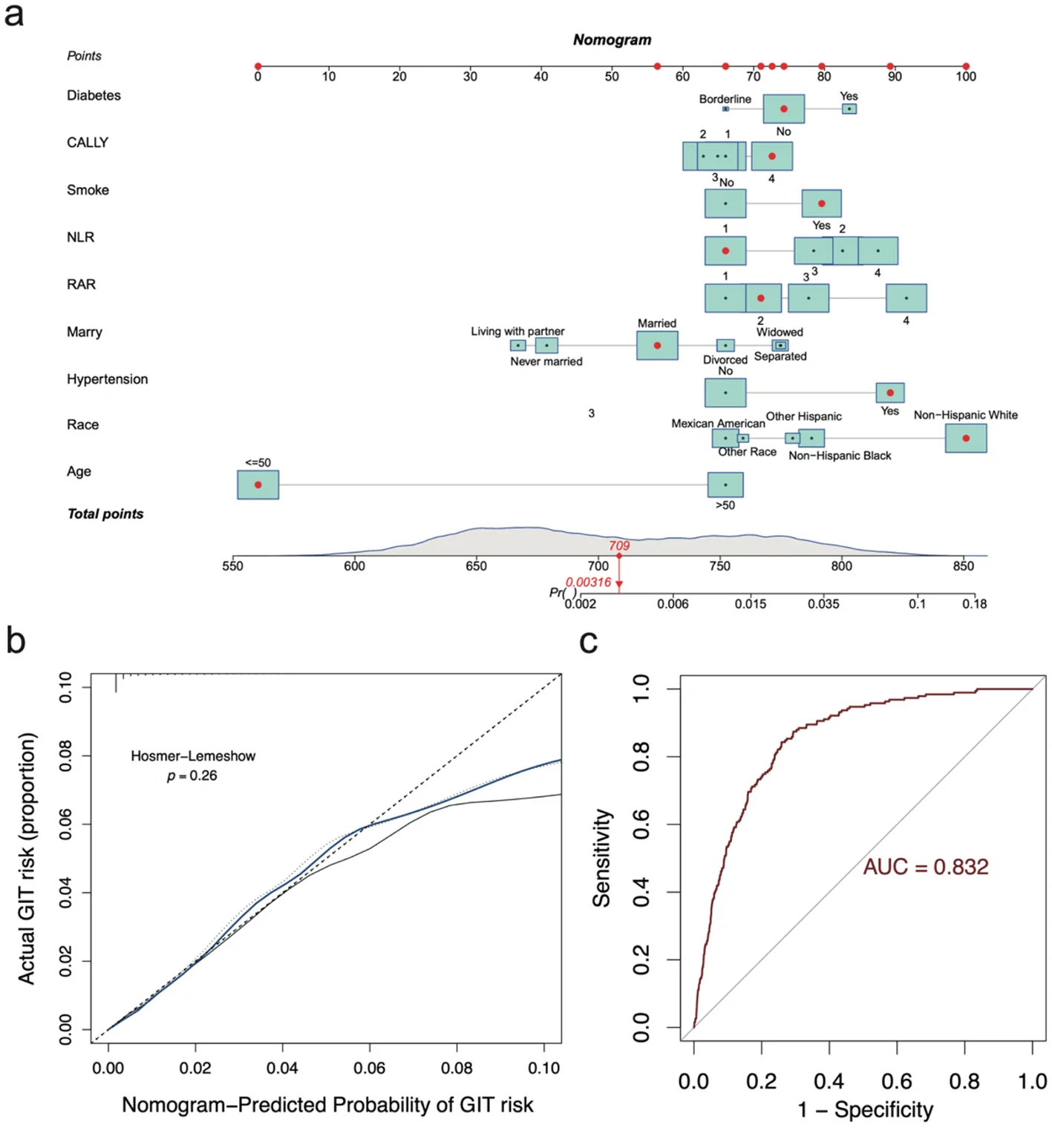
Nomogram for predicting gastrointestinal tumor status and its performance. **(a)** Nomogram incorporating CALLY, NLR, RAR and clinical variables to estimate tumor status; **(b)** Calibration curve; **(c)** ROC curve.

A nomogram integrating CALLY, NLR, RAR, and relevant clinical variables was constructed to distinguish individuals with GIT from controls, with LMR excluded from the final model due to multicollinearity arising from shared components among inflammatory indices (Figure 4a). The calibration curve demonstrated good agreement between the predicted and observed probabilities, and the Hosmer–Lemeshow test indicated good calibration (Figure 4b). In addition, ROC analysis showed that the model achieved an AUC of 0.852, indicating a good ability to discriminate gastrointestinal tumor cases from healthy controls (Figure 4c). Overall, these results suggest that the inflammation-based nomogram has good discriminative value for distinguishing individuals with GIT from controls.

### 3.6 Assessment of the impact of inflammatory markers on the prognosis of GIT patients

Survival analysis was further performed among 191 participants with gastrointestinal tumors in NHANES, including 121 deceased patients and 70 surviving patients during follow-up. The proportional hazards assumption was first assessed for each inflammatory index, and the results are shown in Supplementary Table 1. In subsequent Cox proportional hazards regression analyses, several inflammatory indices were associated with all-cause mortality among patients with GIT. In particular, higher quartiles of RAR were consistently associated with increased mortality risk across the progressively adjusted models. The detailed HRs, 95% CIs, and *P* values from the Cox regression models are presented in Supplementary Figure 4.

**Figure 5:**
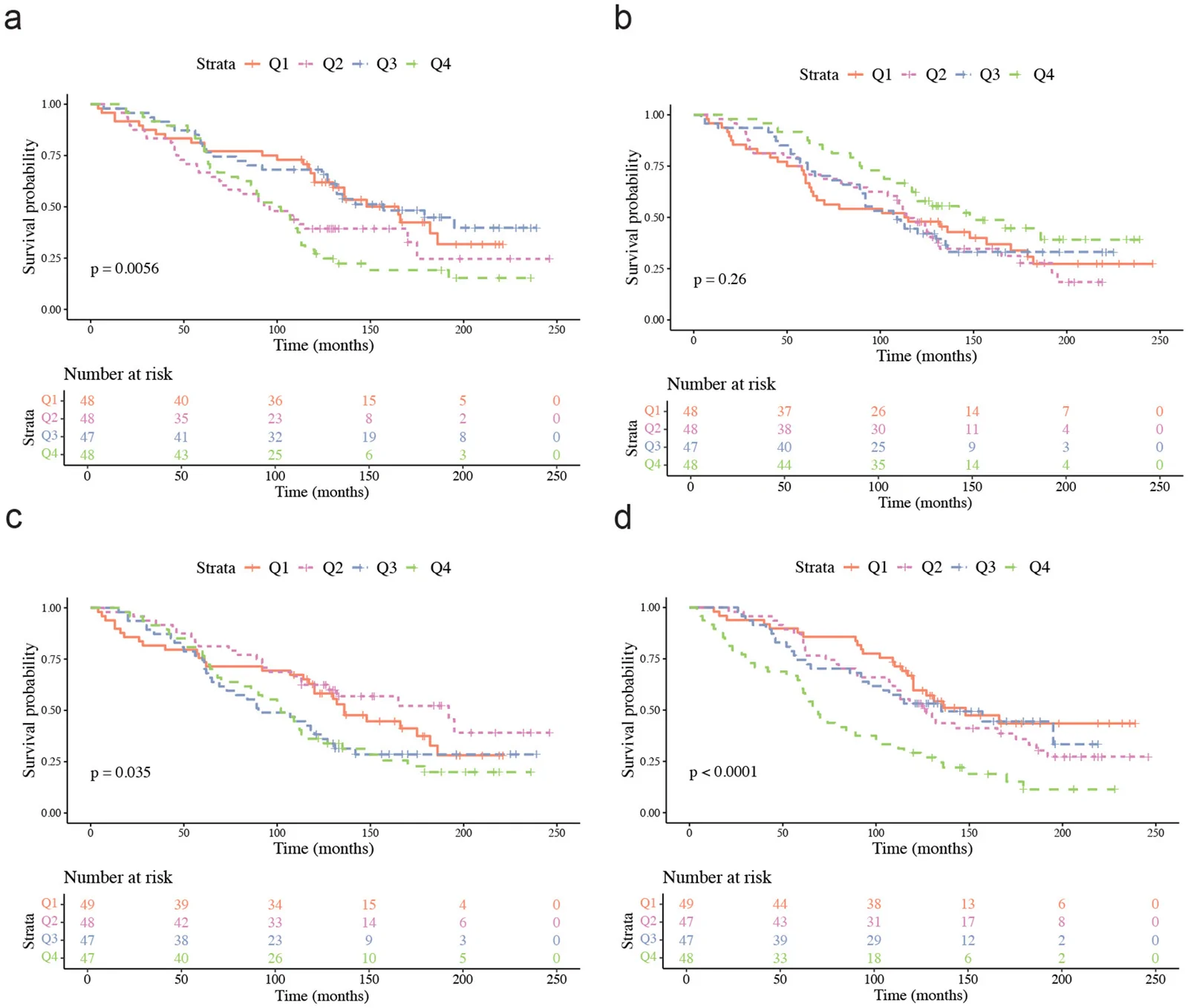
Kaplan-Meier survival curves according to inflammatory index quartiles in patients with GIT. Kaplan-Meier survival curves for patients with GIT stratified by quartiles of selected inflammatory indices: **(a)** SIRI, **(b)** CALLY, **(c)** NAR, and **(d)** RAR.

Kaplan-Meier survival analyses were further conducted for inflammatory indices that remained statistically significant in the fully adjusted Cox regression model (Figure 5). Specifically, SIRI, CALLY, NAR, and RAR were selected for survival curve analysis because they showed significant associations with all-cause mortality in Model 3. Significant survival differences were observed across quartile groups of SIRI, NAR, and RAR, with log-rank *P* values of 0.0056, 0.035, and < 0.0001, respectively, whereas CALLY did not show a statistically significant survival difference across quartiles (*P* = 0.26).

### 3.7 Validation of inflammatory indices in the hospital-based cohort

**Figure 6:**
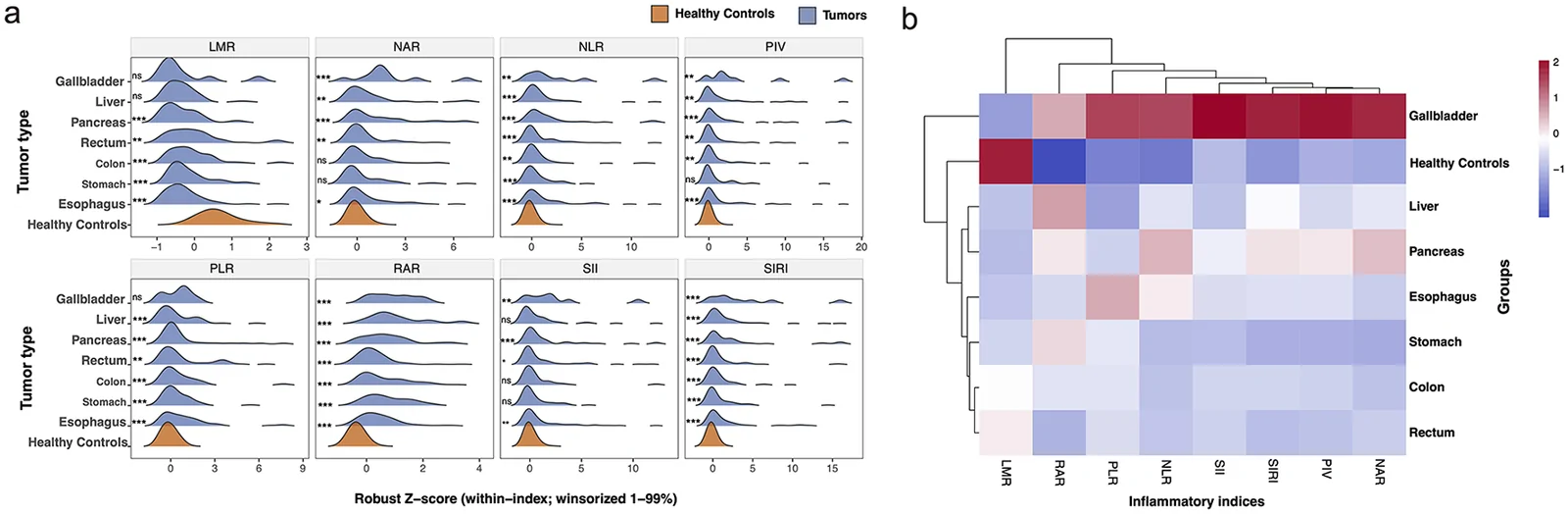
Distribution patterns of systemic inflammatory indices across different digestive system tumors in the hospital-based cohort. **(a)** Ridgeline density plots showing the distribution of eight inflammatory indices among healthy controls and different tumor types. ns, not significant; *, *P* < 0.05; **, *P* < 0.01; ***, *P* < 0.001. **(b)** Heatmap illustrating the relative levels and clustering patterns of inflammatory indices across tumor types.

To further validate the findings from the NHANES dataset, an independent hospital-based cohort was analyzed. The baseline clinical characteristics and inflammatory indices of this cohort are summarized in Table 2. Overall, several inflammatory markers, including NLR, PLR, RAR, SII, SIRI, and PIV, tended to show higher levels in tumor groups than in healthy controls, whereas LMR generally exhibited lower levels among tumor patients, indicating an inflammatory pattern similar to that observed in the population-based analysis (Figure 6a).

**Table 2:** Baseline clinical characteristics and inflammatory indices of the hospital-based validation cohort.

Variable	Level	Control	GIT	*P*
Gender (%)	Female	98 (46.0)	80 (35.2)	0.028
	Male	115 (54.0)	147 (64.8)	
Age (%)	>50	94 (44.1)	216 (95.2)	< 0.001
	≤ 50	119 (55.9)	11 (4.8)	
SIRI		0.65 (0.45, 0.99)	1.08 (0.67, 2.38)	< 0.001
RAR		0.28 (0.26, 0.30)	0.36 (0.32, 0.44)	< 0.001
NLR		1.77 (1.37, 2.44)	2.53 (1.69, 4.55)	< 0.001
PLR		116.67 (90.11, 151.46)	148.18 (107.58, 230.79)	< 0.001
LMR		5.50 (4.48, 6.65)	2.75 (1.88, 4.17)	< 0.001
SII		399.61 (282.15, 576.99)	473.18 (297.62, 1,091.47)	0.002
PIV		144.11 (91.05, 227.58)	203.01 (112.30, 480.27)	< 0.001
NAR		0.07 (0.06, 0.09)	0.09 (0.06, 0.14)	0.004

In addition to gastrointestinal tract tumors, we also analyzed hepatobiliary–pancreatic cancers, including liver, gallbladder, and pancreatic tumors. The heatmap analysis further illustrated the relative levels of inflammatory indices across different tumor types (Figure 6b). Notably, distinct inflammatory profiles were observed between gastrointestinal tract cancers and hepatobiliary–pancreatic cancers. In particular, gallbladder cancer showed the most pronounced inflammatory pattern overall. Hierarchical clustering showed that esophageal, gastric, colon, and rectal cancers clustered together, while liver and pancreatic cancers formed another cluster, highlighting distinct inflammatory profiles across tumor types. These findings provide external validation supporting the association between systemic inflammatory indices and digestive system tumors.

### 3.8 Multivariable logistic regression analysis in the hospital-based cohort

**Figure 7:**
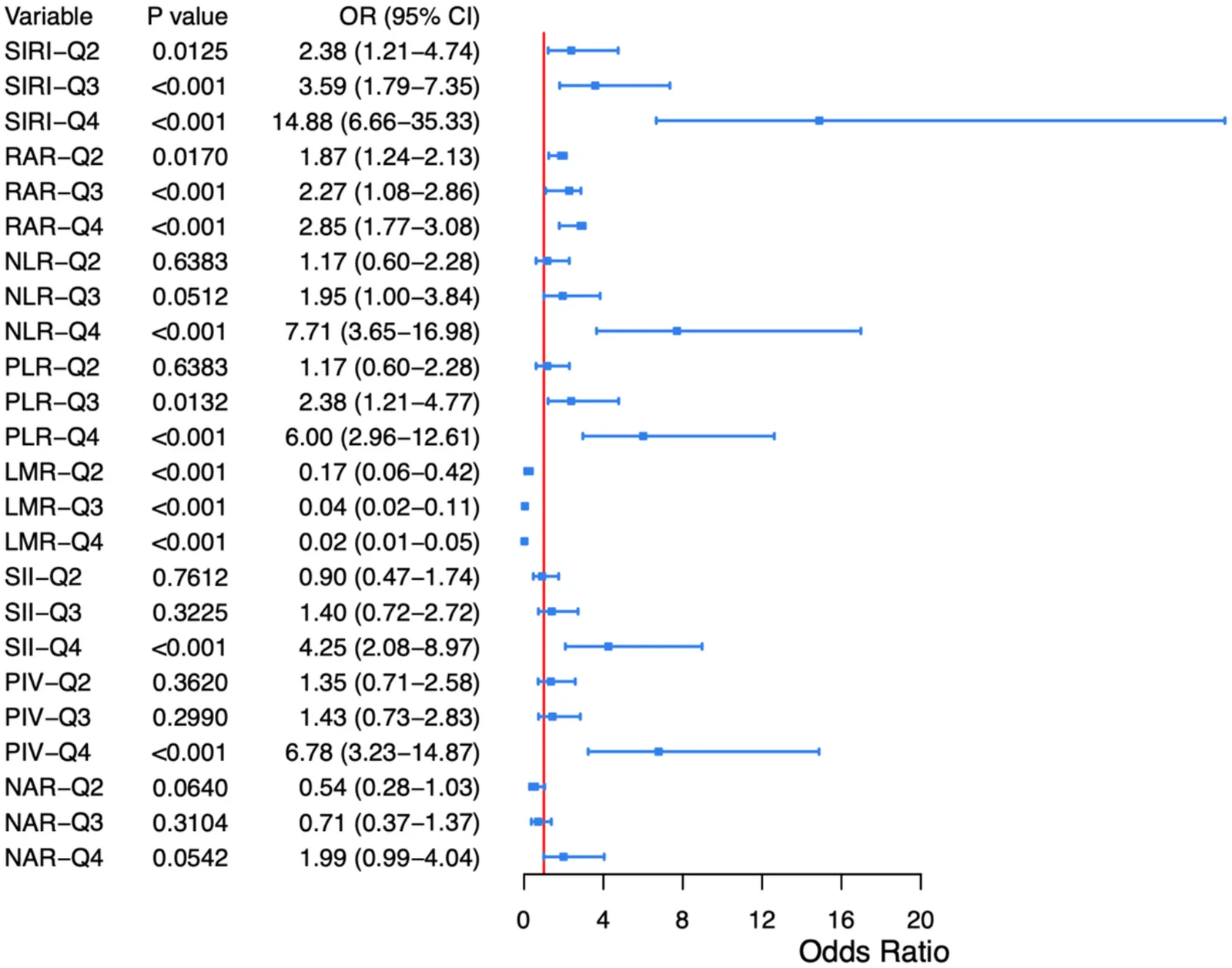
Multivariable logistic regression analysis of systemic inflammatory indices in the hospital-based cohort.

In the hospital-based cohort, multivariable logistic regression analyses adjusted for age and sex further validated the associations between several systemic inflammatory indices and gastrointestinal tumors (Figure 7). Higher quartiles of SIRI, RAR, NLR, PLR, and PIV were associated with increased odds of GIT, whereas elevated LMR levels were inversely associated with gastrointestinal tumors. Among these indices, RAR and SIRI showed particularly strong associations across increasing quartiles. These findings were generally consistent with the NHANES results and further supported the potential diagnostic value of systemic inflammatory markers in gastrointestinal tumors.

### 3.9 Construction and validation of the composite inflammation score

**Figure 8:**
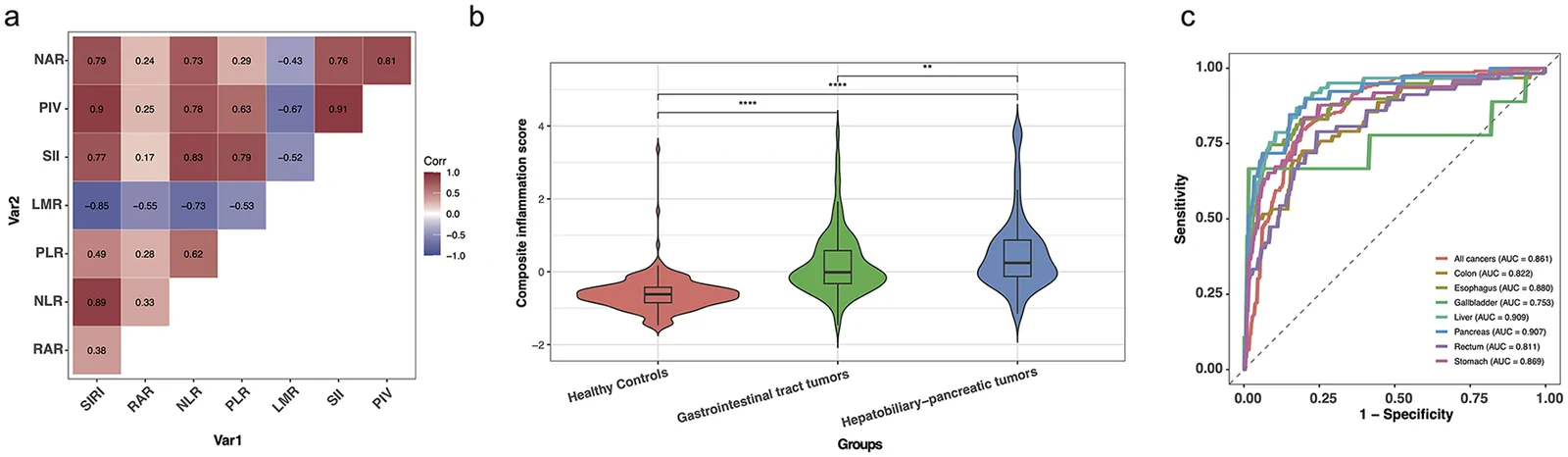
Composite inflammation score and its discriminative performance. **(a)** Correlation analysis of inflammatory indices. **(b)** Distribution of the composite inflammation score. **(c)** ROC curve.

Correlation analysis revealed substantial associations among several inflammatory indices, suggesting potential redundancy among variables (Figure 8a). Based on these correlations, five representative inflammatory markers (RAR, NLR, PLR, LMR, and NAR) were selected for the subsequent construction of the composite inflammation score using principal component analysis. When the composite inflammation score was compared across groups, both gastrointestinal tract tumors and hepatobiliary–pancreatic tumors exhibited significantly higher scores than healthy controls, with hepatobiliary–pancreatic tumors showing relatively higher inflammatory scores overall (Figure 8b). Further ROC analysis demonstrated that the composite inflammation score had good discriminatory ability for detecting digestive system cancers (Figure 8c).

## 4 Discussion

In this study, we systematically evaluated the associations between multiple systemic inflammatory indices and gastrointestinal tumors using population-based data from NHANES. Several inflammatory markers showed significant associations with the presence of gastrointestinal tumors. In particular, higher levels of indices reflecting systemic inflammation, such as NLR, RAR, and SIRI, were associated with increased odds of gastrointestinal tumors, whereas indices related to immune status, including LMR and CALLY, showed inverse associations. In addition to conventional regression analyses, machine-learning models were applied to identify the most informative inflammatory markers, and restricted cubic spline analyses further showed nonlinear association patterns between several indices and the presence of gastrointestinal tumors. Moreover, analyses based on an independent hospital-based cohort showed similar distribution patterns of inflammatory markers across tumor groups, supporting the robustness of the observed associations. Together, these findings suggest that systemic inflammatory imbalance may be closely associated with gastrointestinal tumor status.

Chronic inflammation is considered an important driving factor in tumor development and progression (17, 18). Persistent inflammatory responses can promote carcinogenesis through several mechanisms, including increased oxidative stress, DNA damage, and alterations in the tumor microenvironment (19–21). Inflammatory cells such as neutrophils and monocytes can release cytokines, chemokines, and reactive oxygen species that facilitate tumor growth, angiogenesis, and metastatic potential (22, 23). At the same time, systemic inflammation may suppress effective antitumor immune responses (24), thereby creating a microenvironment that favors tumor progression. Because peripheral blood inflammatory indices reflect the balance between inflammatory activation and immune regulation, they have been increasingly investigated as accessible biomarkers related to cancer status and clinical outcomes (25).

Among the inflammatory markers examined in this study, several indices showed particularly strong associations with GIT. NLR, which reflects the balance between neutrophil-mediated inflammation and lymphocyte-mediated immune response, has been widely reported as a marker associated with tumor development and prognosis in multiple cancers (26, 27). Elevated neutrophil levels may promote tumor progression through the secretion of pro-inflammatory cytokines and growth factors, whereas reduced lymphocyte levels may indicate weakened antitumor immune surveillance. In contrast, LMR represents the balance between lymphocytes and monocytes. Lower LMR values may reflect both impaired immune function and increased monocyte-related inflammatory activity, which may contribute to tumor-promoting microenvironments (28, 29). In addition, RAR integrates information from red cell distribution width and serum albumin, thereby linking systemic inflammation with nutritional and metabolic status (30). These indices together may provide complementary information regarding the systemic inflammatory and immune conditions associated with gastrointestinal tumor development.

Among the nine inflammatory indices evaluated in this study, not all markers showed significant associations with gastrointestinal tumors. While indices such as NLR, RAR, and LMR demonstrated clearer associations with gastrointestinal tumor status, others showed weaker or non-significant associations. These differences may be partly explained by the biological information captured by each index. NAR showed no significant association in the present analysis. Although NAR also incorporates neutrophil counts, it replaces lymphocyte counts with albumin levels, which primarily reflect nutritional status rather than immune response (31). As a result, NAR may be less sensitive in capturing the immune–inflammatory imbalance that characterizes tumor development. However, this finding is based on the NHANES dataset and requires further validation in independent cohorts. Similarly, PLR did not demonstrate as strong an association as some other indices. Platelet levels can be influenced by multiple physiological conditions, including transient inflammatory responses, metabolic status, and medication use (32). Therefore, PLR may be less specific in reflecting tumor-related inflammatory processes in population-based analyses. These findings suggest that although systemic inflammatory indices share similar components, their biological interpretations and discriminative value for gastrointestinal tumors may differ. Evaluating multiple indices simultaneously may therefore provide a more comprehensive understanding of systemic inflammation in relation to gastrointestinal tumor status.

In addition, we developed a multivariable nomogram incorporating selected inflammatory indices and clinical variables to discriminate individuals with gastrointestinal tumors from controls. The model demonstrated good discriminative ability and calibration performance in the present study. However, because the model was developed using retrospective data, prospective multicenter studies and independent external validation are still required before its potential clinical application. By integrating multiple complementary indicators, the nomogram may provide a more intuitive approach for evaluating inflammation-related differences between individuals with and without gastrointestinal tumors.

The findings from the hospital-based cohort were largely consistent with those observed in the NHANES analysis. In addition to this consistency, we further observed distinct differences in the magnitude of inflammatory responses across tumor types in the local cohort. In the local dataset, hepatobiliary–pancreatic tumors, including liver, pancreatic, and gallbladder cancers, generally exhibited higher composite inflammation scores than tumors arising from the gastrointestinal tract, such as colorectal or gastric cancers. This pattern may reflect differences in the biological background of these malignancies. Hepatobiliary–pancreatic tumors often develop in the context of chronic inflammatory conditions, such as chronic hepatitis, liver cirrhosis, pancreatitis, or biliary tract inflammation (33–35). These conditions are characterized by persistent immune activation and systemic inflammatory responses, which may lead to elevated levels of circulating inflammatory markers. In contrast, tumors originating from the digestive tract may induce more localized inflammatory reactions within the tumor microenvironment, while the systemic inflammatory response detected in peripheral blood may be relatively less pronounced. These observations further support the notion that tumors arising from different organs within the digestive system may present heterogeneous systemic inflammatory profiles.

The present study has several strengths. First, the analysis was based on a large, nationally representative dataset from NHANES, which enhances the generalizability of the findings. Second, unlike most previous studies that focused on a single inflammatory marker, we systematically evaluated multiple inflammation-related indices within the same population, providing a more comprehensive assessment of systemic inflammatory status in gastrointestinal tumors. Third, several complementary analytical approaches, including machine-learning models, restricted cubic spline analyses, nomogram development, and principal component analysis, were applied to characterize the relative importance, nonlinear association patterns, and overall inflammatory profiles of these indices. Finally, an independent hospital-based cohort was used to externally validate the NHANES findings and further investigate inflammatory heterogeneity across different digestive system tumors, thereby strengthening the robustness and clinical relevance of our results.

Cancer status in NHANES was based on self-reported physician diagnoses rather than pathology-confirmed records. This may introduce recall bias and disease misclassification. However, previous studies have shown that self-reported diagnoses of major chronic diseases, including cancer, generally have acceptable agreement with medical records in large population-based surveys (36). Therefore, although some degree of misclassification may exist, it is more likely to weaken the observed associations than to create spurious positive findings.

This study has several limitations. First, the cross-sectional design of the NHANES analysis limits the ability to establish temporal or causal relationships between systemic inflammatory indices and gastrointestinal tumors. Second, the diagnosis of gastrointestinal tumors in NHANES was based on self-reported medical history, which may introduce recall bias or misclassification. Third, detailed clinical information regarding recurrence status, metastatic disease, previous antitumor treatment, disease history, and several metabolic or inflammatory covariates was not consistently available in the hospital-based cohort. In addition, including some additional variables in the NHANES analysis would have substantially reduced the number of eligible GIT cases because of missing data. These factors may influence systemic inflammatory indices and should therefore be considered when interpreting the findings. Fourth, the nomogram model was constructed and evaluated retrospectively, and external validation in larger multicenter cohorts is still required before potential clinical application. Finally, the hospital-based cohort was derived from a single center, and the sample size for several tumor subtypes remained relatively limited, which may affect the generalizability and stability of subgroup analyses. Future prospective and multicenter studies are needed to further validate these findings and clarify the biological mechanisms underlying the observed associations.

## 5 Conclusion

In conclusion, several systemic inflammatory indices were significantly associated with gastrointestinal tumors in the NHANES population, suggesting that routine blood-based markers may reflect systemic immune alterations related to tumor presence. These findings were further supported by the hospital-based cohort, which validated the association between elevated inflammatory levels and gastrointestinal tumors. Notably, hepatobiliary–pancreatic tumors tended to exhibit higher inflammation scores compared with tumors originating from the gastrointestinal tract, highlighting potential differences in tumor-associated immune responses. In addition, a nomogram integrating key inflammatory indices demonstrated good discriminative performance, indicating its potential value for distinguishing individuals with gastrointestinal tumors from controls. Furthermore, elevated baseline inflammatory indices were associated with increased all-cause mortality after multivariable adjustment, suggesting their potential application in individualized risk assessment. Overall, these results underscore the value of systemic inflammatory markers in understanding tumor-related immune changes and may provide useful information for identifying inflammation-associated profiles and prognostic risk in gastrointestinal cancers.

## Data Availability

The raw data supporting the conclusions of this article will be made available by the authors, without undue reservation.
