# Supplementary material for: Association of systemic inflammatory indices with gastrointestinal tumors: evidence from NHANES and a hospital-based cohort
Source: Front Med (Lausanne). 2026 Aug 10;13:1886964. doi: 10.3389/fmed.2026.1886964 (PMC13500251; doi:10.3389/fmed.2026.1886964)
Supplement: Supplementary file 1 [file Table_1.docx]

| **chisq** | **df** | **p** | **group** |
| --- | --- | --- | --- |
| 3.743 | 3 | 0.291 | SIRI |
| 3.980 | 3 | 0.264 | CALLY |
| 4.350 | 3 | 0.226 | RAR |
| 1.731 | 3 | 0.630 | NLR |
| 5.866 | 3 | 0.118 | PLR |
| 2.153 | 3 | 0.541 | LMR |
| 0.459 | 3 | 0.928 | SII |
| 3.621 | 3 | 0.305 | PIV |
| 0.868 | 3 | 0.833 | NAR |

**Supplementary Table 1**. Proportional hazards assumption test for inflammatory indices in patients with gastrointestinal tumors

**Supplementary Figure 1**. Machine-learning model performance and SHAP-based feature importance in the balanced cohort

Machine-learning analysis based on the balanced 1:1 matched dataset. (a) ROC)curve comparison of different machine-learning models for distinguishing participants with gastrointestinal tumors from controls. (b) SHAP value distribution plot showing the contribution of each inflammatory index to model prediction.

**Supplementary Figure 2**. Multivariable logistic regression analysis after propensity score matching

Forest plots showing the associations between quartiles of systemic inflammatory indices and gastrointestinal tumors in the propensity score-matched cohort. ORs and 95% CIs were estimated across three progressively adjusted models. (a) Model 1; (b) Model 2; (c) Model 3.

**Supplementary Figure 3**. Subgroup analyses of the associations between inflammatory indices and gastrointestinal tumors

Forest plots showing exploratory subgroup analyses stratified by sex, age, smoking status, and alcohol consumption for the associations between inflammatory indices and gastrointestinal tumors. These subgroup analyses were performed without additional covariate adjustment and were used to describe crude association patterns within each subgroup. Panels show results for (a) CALLY, (b) LMR, (c) NLR, and (d) RAR.

**Supplementary Figure 4**. Cox regression analysis of systemic inflammatory indices and all-cause mortality in patients with gastrointestinal tumors

Forest plots showing the associations between quartiles of systemic inflammatory indices and all-cause mortality among patients with gastrointestinal tumors. HRs and 95% CIs were estimated using Cox proportional hazards regression models across three progressively adjusted models. (a) Model 1; (b) Model 2; (c) Model 3.
